# Implementation contextual factors related to community-based active travel to school interventions: a mixed methods interview study

**DOI:** 10.1186/s43058-021-00198-7

**Published:** 2021-08-26

**Authors:** MacKenzie Koester, Carolina M. Bejarano, Ann M. Davis, Ross C. Brownson, Jon Kerner, James F. Sallis, Chelsea Steel, Jordan A. Carlson

**Affiliations:** 1 Center for Children’s Healthy Lifestyles and Nutrition, Children’s Mercy Kansas City and University of Kansas Medical Center, Kansas City, Missouri USA; 2 Clinical Child Psychology Program, University of Kansas, Lawrence, Kansas USA; 3 Department of Pediatrics, University of Kansas Medical Center, Kansas City, Kansas USA; 4 Prevention Research Center in St. Louis, Brown School, Washington University in St. Louis, St. Louis, Missouri USA; 5 Division of Public Health Sciences, Washington University School of Medicine, Washington University in St. Louis, St. Louis, Missouri USA; 6 Canadian Partnership Against Cancer, Toronto, Ontario Canada; 7 Herbert Wertheim School of Public Health and Human Longevity Science, University of California, San Diego, La Jolla, California USA; 8 Mary MacKillop Institute for Health Research, Australian Catholic University, Melbourne, Australia; 9 Department of Pediatrics, Children’s Mercy Kansas City and University of Missouri Kansas City, Kansas City, Missouri USA

**Keywords:** Barriers, Children, Consolidated framework for implementation research, Facilitators, Physical activity

## Abstract

**Background:**

Active travel to school contributes to multiple physical and psychosocial benefits for youth, yet population rates of active travel to school are alarmingly low in the USA and many other countries. Though walking school bus interventions are effective for increasing rates of active travel to school and children’s overall physical activity, uptake of such interventions has been low. The objective of this study was to conduct a mixed methods implementation evaluation to identify contextual factors that serve as barriers and facilitators among existing walking school bus programs.

**Methods:**

Semi-structured interviews guided by the Consolidated Framework for Implementation Research (CFIR) were conducted with leaders of low-sustainability (*n =* 9) and high-sustainability (*n =* 11) programs across the USA. A combination of quantitative (CFIR-based) coding and inductive thematic analysis was used. The CFIR-based ratings were compared between the low- and high-sustainability programs and themes, subthemes, and exemplary quotes were provided to summarize the thematic analysis.

**Results:**

In both the low- and high-sustainability programs, three of the 15 constructs assessed were commonly rated as positive (i.e., favorable for supporting implementation): *student/family needs and resources*, *implementation climate*, and *planning*. Three constructs were more often rated as positive in the high-sustainability programs: *organizational incentives and rewards*, *engaging students and parents*, and *reflecting and evaluating*. Three constructs were more often rated as positive in the low-sustainability programs: *student/family needs and resources - built environment, available resources,* and *access to knowledge and information*. Four themes emerged from the thematic analysis: *planning considerations, ongoing coordination considerations, resources and supports*, and *benefits.*

**Conclusions:**

Engagement of students, parents, and community members were among the factors that emerged across the quantitative and qualitative analyses as most critical for supporting walking school bus program implementation. The information provided by program leaders can help in the selection of implementation strategies that overcome known barriers for increasing the long-term success of community-based physical activity interventions such as the walking school bus.

## Introduction

Walking and/or cycling to and/or from school is associated with higher overall physical activity among children, accounting for about 16 min of a child’s recommended 60 min of daily physical activity, and can contribute to many physical and psychosocial health benefits for youth [1, 2]. However, only about 11% of youth in the USA engage in active travel to school, and rates are similarly low in many other countries [3].

Active travel to school among youth is low due to multiple environmental and psychosocial barriers. Distance to school has been identified as a primary barrier to participating in active travel to school [4, 5]. However, even when children live within walking distance of their school, active travel to school rates remains low [4, 5]. Aside from distance, lack of safety from traffic and crime are the main environmental barriers, while parental concerns/attitudes and lack of social support are the main psychosocial barriers [6–11].

Walking school bus programs are a form of active travel to school and are defined as an organized group of children walking to and/or from school, usually under the supervision of adult chaperones [12]. Walking school bus programs have been shown to increase overall physical activity and provide health benefits to youth [13, 14]. These programs help address many of the aforementioned environmental and psychosocial barriers to walking to school. For example, safety among youth is improved when walking in a group accompanied by an adult, and social support is provided [7].

Despite the positive impact of walking school bus programs on active travel and physical activity, only about 6% of all USA elementary schools are reported to have an active walking school bus program [15]. Research indicates that implementation success and sustainability are challenging among existing walking school bus programs [16]. A broader body of literature has pointed to the critical role of context in the uptake and implementation of evidence-based interventions in real-world settings [17, 18]. A better understanding of implementation contextual factors that serve as barriers and facilitators to success is critical in informing implementation efforts of such interventions, including for supporting more widespread adoption and sustainability of walking school bus programs across the USA and other parts of the world where rates of active travel to school are low. It is particularly important to improve understanding of contextual factors that differentiate more- from less-sustainable programs to support successful implementation models.

The objective of the present study was to conduct a mixed methods implementation evaluation to identify contextual factors among existing low- and high-sustainability walking school bus programs in the USA. The interviews and quantitative analyses were guided by the Consolidated Framework for Implementation Research (CFIR) [19], and inductive thematic analysis was used to provide additional depth to the interview data [20].

## Methods

### Participants and procedures

Participants included 20 walking school bus program coordinators (key informants) who were recruited from a larger study of existing walking school bus programs in the USA and other countries [21]. Participants for the larger study were identified and recruited through multiple sources, including Internet searches and indirectly through intermediary contacts (e.g., pedestrian organizations, health departments). Key informants (*N*=145) representing 184 walking school bus programs completed the larger study in 2017–2018. The 110 key informants from programs located within the USA were considered eligible for the interview subsample, with data collection occurring in 2018–2019. The institutional review board approved the recruitment and procedures for the interview study.

Survey participants were stratified into either a low- or high-sustainability group based on a survey item asking, “What is the likelihood that the walking school bus program at your school will continue next year?” The four-point Likert-scale included the options *extremely unlikely*, *somewhat unlikely*, *somewhat likely*, and *extremely likely*. Those who reported their program was *somewhat* or *extremely unlikely* to continue were categorized as low sustainability, which included 9 key informants. All 9 of these key informants were invited to participate in the interview and 7 were successfully recruited. To identify more low-sustainability programs, the 18 key informants who reported in the survey their program had been in existence ≤1 year (open ended question) were contacted to inquire whether their program was still in operation. Two key informants stated their program had stopped or was struggling to continue and were successfully recruited to participate in the interview as part of the low-sustainability group. Thus, the final low-sustainability group comprised a total of 9 interview participants. The 84 key informants who reported in the survey their program was *extremely likely* to continue were categorized as high sustainability. A random sample of 25 key informants from the high-sustainability group was invited to participate in the interview. Interviews were conducted with the first 11 participants who responded to the interview invitation, with a goal of interviewing an approximately equal number of key informants from each sustainability group. Upon completion of the interview, participants were provided with a $50 e-gift card.

### Program descriptive characteristics

Several items from the survey and the proportion of students eligible for free or reduced-price lunch from public data (an indicator of area income) were used to describe the study sample. Items from the survey included location (region of the USA), program coordinator type (parent, school staff, or external organization staff), receipt of external funding, and program size/scope (number of students, number of trips/week, and number of route leaders) [21]. The data were summarized within the low- and high-sustainability groups to describe the study sample.

### Interview guide and interviews

The CFIR was adapted for the present study to fit the community context and specifically the walking school bus intervention. CFIR consists of 39 constructs used to evaluate barriers and facilitators of program implementation [19]. The constructs capture five domains: intervention characteristics, outer setting, inner setting, characteristics of individuals, and process. The interview guide provided by the CFIR team was adapted for use in the present study [22]. To keep the interview within 1 h, a subset of constructs was selected for inclusion using an iterative process involving multiple discussions among research team members. Constructs believed to be most relevant to walking school bus programs were retained, and refinements were made to item wording to create specificity to the walking school bus. The CFIR patient needs and resources construct within the outer setting domain was renamed student/family needs and resources and split into two sub-constructs, with one sub-construct used to capture the neighborhood-built environment as it related to student/family needs and resources. The CFIR engaging construct within the process domain was split into three sub-constructs to capture engagement with different stakeholders: route leaders, students and parents, and external change agents. The final interview guide covered 15 of the 39 CFIR constructs (see Table 1).

**Table 1:** Definitions and rating criteria for constructs assessed

CFIR domain and construct	Interview question	Negative rating	Positive rating
1. Intervention characteristics: program source	How was the walking school bus program at your school started and who was involved?	External from the school	Internal to the school
2. Intervention characteristics: cost	What financial costs are associated with the walking school bus program?	Program has high costs	Program has low or no costs
3. Outer setting: student/family needs and resources^a^	What needs and preferences of students and parents were considered when planning the walking school bus program, and how did you know about these needs and preferences?	Needs and preferences not taken into account	Needs and preferences taken into account
4. Outer setting: student/family needs and resources - built environment^a^	What features of the neighborhood environment around the school serve as barriers to or facilitators of the walking school buses success?	More barriers than facilitators observed in the built environment	More facilitators than barriers observed in the built environment
5. Inner setting: implementation climate	To what extent do teachers and staff at the school value and support the walking school bus program?	Teachers and school staff not supportive of the program	Teachers and school staff supportive of the program
6. Inner setting: relative priority	To what extent has the walking school bus program had to compete with other priorities or initiatives going on at the school?	walking school bus program has competition	walking school bus program does not have competition
7. Inner setting: organizational incentives and rewards	What kinds of incentives are there for students, parents, and those involved in operating the walking school bus program?	Minimal or no incentives for students, parents, and those involved in program operations	Sufficient incentives for students, parents, and those involved in program operations
8. Inner setting: leadership engagement	To what extent do leaders at the school, such as the principal, value and support the walking school bus program?	School leaders not supportive of the program	School leaders supportive of the program
9. Inner setting: available resources	What level of resources has the school dedicated to the walking school bus Program, and how have these been leveraged?	Minimal or no resources dedicated to the program	Sufficient resources dedicated to the program
10. Inner setting: access to knowledge and information	What kinds of information and materials about operating walking school bus programs (e.g., implementation guides, toolkits, trainings) have been available to you?	Minimal or no information resources available	Sufficient informational resources available
11. Process: planning	What kind of planning is involved in starting, operating, and maintaining the walking school bus?	Minimal or no planning	Sufficient amount of planning
12. Process: engaging route leaders^b^	Describe your process for working with route leaders; how do you recruit, retain, and coordinate with route leaders?	Minimal or no procedures working with route leaders	Sufficient procedures working with route leaders
13. Process: engaging students and parents^b^	How do you recruit students to participate in the walking school bus and maintain their participation?	Minimal or no student recruitment strategy	Sufficient student recruitment strategy
14. Process: engaging external change agents^b,c^	Is someone (or a team) outside your school helping you with planning, coordinating, or implementing the walking school bus program? If so, how?	No Outside organizational help	Outside organizational help
15. Process: reflecting and evaluating	What kind of data do you collect as you implement the walking school bus program?	Minimal or no data collected	Sufficient amount of data collected

^a^The CFIR Student/family needs and resources construct was split into two sub-constructs for the present study

^b^The CFIR Engaging construct was split into three sub-constructs for the present study

^c^Item was only asked in the 7 low-sustainability and 7 high-sustainability programs that were coordinated by a parent or school member

Two study personnel trained in conducting qualitative interviews and in the CFIR framework led the semi-structured interviews. The interviews were conducted over the phone and audio recorded with the participant’s verbal consent. The interviews lasted between 30 min and 1 h. The audio recordings were transcribed verbatim.

### Interview coding and analysis

#### Overview

Both quantitative (CFIR-based) analyses and inductive thematic analyses were employed in interview coding to utilize a mixed-methods approach [23]. The intent of the thematic analysis was to provide a richness and depth of information and to minimize the likelihood of missing insights through only conducting quantitative analysis.

#### Quantitative analysis based on CFIR

Two trained study personnel coded each interview transcript independently, after all interviews were completed. Each rater created a memo summary for each construct and rated its presence and valence (−2 = negative to 2 = positive) using the rating rules established by the CFIR team [24]. The rating rules determine that if a construct appears to have acted to strongly benefit the program, a rating of 2 is given. Similarly, a rating of 1 is given when the construct appears to have been beneficial, but to a lesser degree. Constructs appearing to have acted detrimentally/negatively received a −1 or −2 rating. Constructs not codable or neutral received a 0 rating. Table 1 provides more detail regarding what constituted a positive vs. negative rating for each construct. Each rater entered their independent ratings into an excel spreadsheet. Inter-rater agreement in the form of percent agreement was assessed for each construct after grouping the ratings into 3 categories: negative ratings (−2, −1), neutral or not present (0), and positive ratings [1, 2]. Criteria for interpreting agreement were ≥75% = good to excellent, 60–74% = moderate, and <60% = poor. Grouping the values was necessary to improve rater agreement. After assessing agreement, any rating discrepancies between the two raters were reconciled through verbal discussion. The final construct ratings for each interview were reached with the raters coming to a final consensus. This approach to inter-rater reliability, calculating agreement and basing final ratings on reconciliation, was used to support the validity of the ratings [25]. The final construct ratings were compared between the low- and high-sustainability groups.

#### Inductive thematic analysis

The study team completed an inductive thematic analysis on all interview content to identify themes within the interviews following the procedures of Braun and Clarke [20]. Inductive analysis was chosen over deductive analyses (mapping interview content to the CFIR domains) because the latter was viewed as overly restrictive and likely to result in failure to detect important themes (i.e., themes not clearly covered within the CFIR). Four study personnel, including two study investigators and two masters-level research assistants, independently reviewed five randomly allocated interviews to note potential themes. The research members then met multiple times to discuss and reconcile the final list of themes and create a heading and definition for each theme. Initially, each team member identified between six and 18 themes. The team members then discussed, discarded, and grouped themes through iterative discussion. Once the final themes and definitions were established, one study member conducted data extraction by reviewing all interview transcripts and selecting six to ten quotes that fit each theme. The four team members then independently selected the three quotes they felt best represented each theme. The three to four quotes within each theme that were selected with the highest frequency across the team members were selected for inclusion in this paper to exemplify each theme.

## Results

Characteristics of the included programs are presented in Table 2. The programs varied with regard to geographic location, area economic status, level of support/funding, type of coordinator, and size/scope, indicating that the sample captured heterogeneity in the program models that exist in practice. The largest differences between the low- and high-sustainability programs were that the latter were larger, with more students and more route leaders.

**Table 2:** Descriptive characteristics of the walking school bus programs

	Low-sustainability programs *N*=9	High-sustainability programs *N*=11
Region, *N* (%)		
Northeast	0 (0%)	2 (18%)
Midwest	4 (44%)	5 (46%)
West	3 (33%)	1 (9%)
Southwest	0 (0%)	1 (9%)
Southeast	2 (22%)	2 (18%)
Coordinator, *N* (%)		
Parent	2 (22%)	2 (18%)
School staff	5 (56%)	5 (46%)
External organization	2 (22%)	4 (36%)
External funding, *N* (%)		
Yes	4 (44%)	6 (55%)
No	5 (56%)	5 (45%)
Free or reduced price lunch eligibility, mean (SD)	45% (28%)	55% (33%)
Number of student participants, mean (SD)	28.56 (18.32)	63.64 (120.29)
Number of trips per week, mean (SD)	3.22 (3.49)	3.73 (2.83)
Number of route leaders, mean (SD)	3.11 (3.44)	7.91 (6.35)

### Quantitative interview analysis guided by CFIR

The inter-rater agreement percentages for the initial ratings are reported in Table 3. Nine of the 15 constructs had good-to-excellent agreement (75 to 93%) between raters. Four constructs had moderate agreement (60 to 70%) between raters. Two constructs, *implementation climate* and *engaging route leaders*, had poor agreement (40 to 45%) between raters.

**Table 3:** Proportion of low- and high-sustainability programs with a positive construct rating, by construct

CFIR domain and construct	% agreement between raters^**a**^	***N*** (%) Rated positive^**b**^	
		Low-sustainability programs *N*=9	High-sustainability programs *N*=11
1. Interventions characteristics: program source	75%	3 (33.3%)	4 (36.4%)
2. Intervention characteristics: cost	60%	6 (66.7%)	6 (54.5%)
3. Outer setting: student/family needs and resources	65%	9 (100%)	8 (72.7%)
4. Outer setting: student/family needs and resources - built environment	80%	6 (55.7%)	3 (27.3%)
5. Inner setting: implementation climate	40%	7 (77.8%)	8 (72.7%)
6. Inner setting: relative priority	75%	3 (33.3%)	5 (45.5%)
7. Inner setting: organizational incentives and rewards	90%	5 (55.6%)	9 (81.8%)
8. Inner setting: leadership engagement	85%	6 (66.7%)	8 (72.7%)
9. Inner setting: available resources	70%	7 (77.8%)	5 (45.5%)
10. Inner setting: access to knowledge and information	85%	8 (88.9%)	7 (63.6%)
11. Process: planning	90%	8 (88.9%)	10 (90.9%)
12. Process: engaging route leaders	45%	5 (55.6%)	5 (45.5%)
13. Process: engaging students and parents	70%	5 (55.6%)	11 (100%)
14. Process: engaging external change agents^c^	93%	4 (57.1%)	5 (71.4%)
15. Process: reflecting and evaluating	85%	3 (33.3%)	6 (54.5%)

^a^Based on the initial ratings, which were prior to discrepancies being reconciled

^b^Based on final ratings, which reflect consensus between raters established through reconciliation

^c^Item was only asked in the 7 low-sustainability and 7 high-sustainability programs that were coordinated by a parent or school member

The frequency of positive ratings for each construct was based on the final (reconciled) ratings and is also reported in Table 3. In both the low- and high-sustainability programs, three constructs were commonly (by 72.7 to 100% of programs) rated positively, as being favorable for supporting implementation. These constructs were *student/family needs and resources*, *implementation climate*, and *planning*. Four constructs were moderately commonly (45.5 to 72.7%) rated positively in both the low- and high-sustainability programs: *cost*, *leadership engagement*, *engaging route leaders*, and *engaging external change agents*. The constructs that were least commonly (27.3 to 45.5%) rated positively among both program groups were *program source* and *relative priority*.

In gauging differences between the low- and high-sustainability groups, three constructs were more often rated as positive in the high-sustainability programs as compared to the low-sustainability programs: *organizational incentives and rewards* (81.8% vs. 55.6%), *engaging students and parents* (100% vs. 55.6%), *and reflecting and evaluating* (54.5% vs. 33.3%). Three constructs were more often rated as positive in the low-sustainability programs as compared to the high-sustainability programs: *student/family needs and resources - built environment* (55.7% vs. 27.3%), *available resources* (77.8% vs. 45.5%), and *access to knowledge and information* (88.9% vs. 63.6%).

### Qualitative thematic analysis

Four themes emerged from the qualitative analyses: planning considerations, ongoing coordination considerations, resources and supports, and benefits. A summary of each theme, subtheme headings, and exemplary quotations for each subtheme are presented below. Quotations were lightly edited for clarity and confidentiality. LS and HS indicate quotes from low-sustainability and high-sustainability programs, respectively.

#### Planning considerations

Planning activities and related considerations were often noted as being important to successful implementation, either facilitating or hindering a walking school bus program. The planning activities and considerations included buy-in from school administrators, recruiting route leaders, mapping routes, selecting pickup/drop-off sports, scheduling times of operation, addressing neighborhood environment barriers, recruiting students, and gaining trust from parents.

#### Planning considerations: Addressing neighborhood environment barriers

“We went through the National Safe Routes to School surveying process, where we surveyed students, parents, and teachers to kind of ask about what their attitudes were regarding walking and biking, safety, bringing up specific issues with the known neighborhoods or around the schools that may or may not encourage them to walk or bike.” (HS).

#### Planning considerations: Gaining trust from parents

“I think especially for us, where we started off as a new school, just setting the norms that walking or biking to school is something that everybody can and should do. I think just being able to Google and search and find information to help parents feel comfortable with it was really the key to the success. At first, parents were just kind of like, I don’t know. And they were kind of nervous about it. And now it’s just the norm that people don’t even think about.” (HS).

#### Planning considerations: Recruiting students

“So, they get the flyers at the beginning of the year, the principal talks about, you know, walking school bus on the announcements. Because the kids always have something that clips to their backpack that shows they did the walking school bus, I think that kind of generates some excitement.” (HS).

#### Planning considerations: Recruiting route leaders

“At our planning meetings, we would ask if anybody in the meeting knew of folks who might be interested. On the parent surveys that we sent out, we asked if parents would be interested. We did do newspaper articles and we did print flyers for around town. But again, it’s that personal aspect that I think really helped find our volunteers. Oh, and also we put it in the school newsletter. I think that’s how we got some of the staff involved.” (LS).

#### Ongoing coordination considerations

Once a walking school bus was initiated, ongoing coordination activities and related considerations were commonly mentioned regarding facilitating or hindering a walking school bus program. Examples of ongoing coordination activities and related considerations were identifying a coordinator, incentivizing route leader involvement, incentivizing student involvement, accommodating scheduling conflicts or adverse weather, communication among route leaders, communication with parents, communicating program benefits, paperwork/tracking, facilitating transitions in staff, and starting the program back up after school breaks.

#### Ongoing coordination considerations: Incentivizing student involvement

“And I think it was in the spring of ’16, we did a celebrity guest, or like a special guest. And so the kids didn’t know -- on a specific route, who was going to show up for their special guest day. And we did like the firefighter, we did a police officer, we did a football player. A lot of different people that the kids kind of idolize in the community. Plus, it also offered an opportunity for them to learn a little bit more about our program.” (HS).

#### Ongoing coordination considerations: Communication with parents

“We do have a Facebook page that we use for communication to the parents and to the public. I use the remind system, our school district uses that for messaging to parents. So I have a remind group for our walking school bus volunteers and parents, in case there is a day of cancellation, I usually put that on our Facebook page and then send out a text, because not all people have social media. So, I’ve learned I had to communicate in various different ways.” (HS).

#### Ongoing coordination considerations: Scheduling conflicts or adverse weather

“And then just throughout the semester, kind of the day to day. If there’s weather issues we have -- usually we’ll walk in rain, and if there’s lightning or anything like that, use a radar app to look for that. So kind of planning on a day to day depending on weather.” (HS).

#### Ongoing coordination considerations: Communication among route leaders

“I don’t want to take any more of their time with face to face meetings, so a lot of them are great with the remind messages. Some of them don’t have kids in the school district, so they don’t know when days are off, so I communicate that with them through the remind system, through our Facebook page’s communication, and email. And face to face, if, like I’m on a route and I see them, and I ask them how volunteering’s going. If they have any questions, concerns. So, it’s kind of nice that I walk as well.” (HS).

#### Resources and supports

Participants reported that the availability or lack of availability of certain resources and supports can facilitate or hinder a walking school bus program. Examples of resources and supports included funding and costs, safety-related materials, access to student information, support from school administrators, support from school-staff, support from external organizations, support from parents or other community members, and a supportive neighborhood environment.

#### Resources and supports: Costs

“And we’re actually spending money at the school on the kids. A lot of the money goes towards curriculum stuff. Money will go towards incentive items and encouragement items. Recently, we’ve had a lot of interest in neighborhood signage, place making and interventions around our schools and neighborhoods. In some cases, money will go towards us putting together volunteer events where we’ll do kind of clean up, just snow removal events. It altered every year, but I guess what our steady expenses are, there’s always a line in our budget for the safety lessons. There’s always lines in our budget for promotion. There’s always a line for incentive items to promote the programs.” (HS).

#### Resources and supports: Costs

“There’s no financial cost at this time, because we have all of the safety stuff that we need, as far as signage and vests and that kind of thing. The only thing would be the snacks for the kickoffs, and that’s the only thing that we have to pay for. And then if we wanted to give incentives, that would cost money, too, but right now there is nothing coming out of the budget other than for those snacks.” (HS).

#### Resources and supports: Support from parents or other community members

“I think that was the key to the success was having the PTO [parent teacher organization] supportive and pushing the administration of the school and saying that we are the parents; this is important to us; this is what we’re going to do. And also using the PTO’s contact list to spread the information as well.” (HS).

#### Resources and supports: Support from external organizations

“But they [schools] just have so much going on, it seems, and their staff is really stretched, especially in our rural schools that they just can’t -- take this on themselves, so that’s really great that we have our position to come in, bring this great program. We have the resources to run it, to coordinate all the volunteers to put together promotion stuff, so it’s very easy on the school end of things.” (LS).

#### Benefits

Walking school bus programs can have many benefits besides improving children’s health and increasing physical activity. Other noted benefits included reduced truancy among students, convenience for parents, social interaction, improved neighborhood cohesion and sense of community, reduced traffic, increased ability to obtain neighborhood-built environment improvements, and improvements in adults’ health and activity.

#### Benefits: Exercise and socializing

“I think it’s 100% chance it’s going to continue here on out, because the families are -- they do value exercise; they do value the community that is created by walking to school. There’re relationships that are built with teachers by walking into the school. And the -- the community support of it, and the physical -- you know, sidewalks, bike lanes.” (HS).

#### Benefits: Neighborhood improvements

“They’re in the process of building the sidewalks for the rest of those streets. Things like the Safe Routes to School program and walking school bus showing demand for these facilities I think has been a boon for them in showing that, hey, this -- even with the lack of facilities, people want to use it. So it showed demand.” (HS).

#### Benefits: Attendance

“Our school is one that has a really high truancy rate, we have real issues with attendance, and so we have about like 25 kids that walk with us somewhat regularly on the bus and I, you know, because we take daily attendance, so last year I was able to compare these students’ attendance rates from the previous school year to the current school year and some of them were missing like 25-30 days or tardy 25-20 days and their attendance improved greatly once they started walking with the walking school bus.” (HS).

#### Benefits: Safety and traffic

“On Wednesday we even had some parents who, because they’re on one side of the road, it was a very busy on their major intersection road, we had parents who would bring the kids in the car to the end of the not busy road and drop them off with that teacher, and then they walked the rest of the way. Which again helped with our traffic of drop offs coming into school. So, every Wednesday, it was like a huge relief for the car line, obviously. And so that was a huge benefit for it, not only the health of the students.” (LS).

## Discussion

Present findings identified key implementation factors that appear important to target for supporting implementation success and sustainability among walking school bus programs. Such factors involve those that are both internal and external to the school and that relate to multiple levels of influence, including intrapersonal, interpersonal, organizational, and community factors [26]. Taking into account both the quantitative and qualitative interview analyses, engagement of students, parents, and community members were among the factors that emerged as most critical for supporting walking school bus program implementation. Specific engagement strategies were highlighted by respondents in relation to each stakeholder and across planning and coordination activities. A careful selection of implementation strategies that overcome known barriers is likely to lead to increases in long-term success of walking school bus programs and ultimately increased population rates of walking to school and overall physical activity in youth [27, 28].

Given that the CFIR was developed for and has been most often used in clinical settings [19, 29], a unique contribution of the present study was the adaptation of the CFIR for a community-based intervention. One difference between the walking school bus and many clinical interventions is that walking school bus implementation relies primarily on volunteers, which creates unique implementation challenges. The adapted CFIR appeared to have utility for improving understanding of contextual factors surrounding the walking school bus, and many of the themes that resulted from the qualitative analysis aligned with the CFIR constructs though were organized differently. The first theme, planning considerations, could be considered to encompass the CFIR constructs student/family needs and resources, planning, engaging route leaders, engaging students and parents, and engaging external change agents. The theme of ongoing coordination considerations appeared to align with organizational incentives and rewards, engaging route leaders, engaging students and parents, and reflecting and evaluating. The third theme, resources and supports, could be considered to encompass student/family needs and resources—built environment, implementation climate, leadership engagement, available resources, and access to knowledge and information. The fourth and final theme, benefits, relates more to program outcomes and co-benefits than to CFIR constructs. While both the CFIR-related and thematic findings point to factors that appear to be important for supporting sustained implementation of walking school bus programs, the thematic findings provided a depth of insight into specific recommendations/activities. Conversely, the CFIR-based findings are beneficial because they can be used to identify promising implementation strategies based on existing CFIR construct—implementation strategy mappings [30]. Thus, the use of both analytic methods, and particularly their integration (i.e., mixed methods), appears promising for informing implementation studies and should be considered in other implementation research.

The CFIR constructs that were commonly rated as positive in both the low- and high-sustainability programs point to factors that may be critical to target for supporting successful implementation of walking school bus programs but that may not be sufficient for supporting sustainability. These factors included student/family needs and resources, implementation climate, and planning. Results from the thematic analysis suggest that strategies for encouraging a positive implementation climate could include assessing, educating, and involving school leadership in the walking school bus to encourage positive attitudes toward active travel to school. Useful planning activities that emerged included recruiting route leaders through multiple sources and addressing student/family needs by creating appropriate routes, scheduling appropriate times of operation, addressing neighborhood environment barriers, and gaining trust from parents through targeted communication and involvement. Planning activities that aim to identify multiple route leaders may be particularly important given previous research has shown that a top characteristic of more sustainable walking school bus programs is having multiple route leaders [21].

The CFIR constructs that were more commonly rated as positive in the high-sustainability than the low-sustainability programs point to factors that may be critical to target for supporting sustainability. These factors included organizational incentives and rewards, engaging students and parents, and reflecting and evaluating. All high-sustainability programs reported having a detailed process for engaging their students and parents. Strategies for engagement that were highlighted included incentivizing students to support recruitment and participation, such as with promotional swag that can be worn on backpacks or special guests to accompany students on walks. Providing incentives and rewards also appear important for adult leaders and volunteers, which could include stipends, recognition, or acquisition of community service hours. These findings align with previous walking school bus research showing the importance of parent involvement in ongoing implementation success [21]. Reflecting and evaluating did not appear to be common in walking school bus programs, but the differences between sustainability groups suggest this construct is likely important to implementation success. Reflecting and evaluating is an important feature of evidence-based public health [31] and may help program leaders and other stakeholders (school leaders, parents) maintain awareness of problems and rapidly create solutions, as well as helping to show program benefits.

Though it was unexpected that some CFIR constructs were more commonly rated as positive in the low-sustainability than high-sustainability programs (student/family needs and resources—built environment, available resources, and access to knowledge and information), these findings may indicate such factors alone are not sufficient for sustainability. For example, the finding that more low-sustainability programs were rated as having available resources and access to knowledge and information could have been due to these programs requiring more resources to operate due to other barriers. It is also possible that although these facilitators were present, they were not being used as effectively in the low-sustainability programs due to other barriers. The lower neighborhood built environment supportiveness in the low-sustainability programs, and overall moderate-to-low built environment supportiveness across all programs, are relatively encouraging findings because they suggest many programs found success even when the neighborhood environment was somewhat unsupportive of walking. It is possible that walking school bus programs were reported as being more likely to be sustained in areas with built environment barriers because such areas have a greater need for the walking school bus due to its ability to mitigate environmental barriers by providing safety in groups and through adult supervision.

The theme of program benefits surfaced in the thematic findings and appears important for supporting increased uptake and sustainment of walking school bus programs. The primary benefits highlighted in the interviews included increased physical activity for students, parents, and staff; increased socialization and sense of community; improved school attendance; and reduced traffic. These reported benefits are consistent with the other research on active school commuting [1, 21, 32] and indicate that walking school bus programs offer valuable contributions to the physical and psychosocial health of the students and adults involved. Many of the benefits noted were indirect benefits that are often overlooked by health professionals but are important to schools and parents, such as neighborhood-related improvements (e.g., walkability and traffic improvements) and school performance metrics (e.g., improvements in attendance). Highlighting such benefits on individual, local, and societal levels may support increases in school and community involvement in walking school bus programs [33].

### Strengths and limitations

A main strength of this study was the innovative use of implementation science theory-driven data collection and quantitative analyses based on the CFIR [19], paired with qualitative thematic analyses that provided depth of information from program leaders’ lived experiences. The inductive thematic analysis, which was chosen due to its flexibility, captured content outside of the CFIR (e.g., benefits) and led to a slightly different organization of information as compared to the organization of the CFIR. While these were viewed as study strengths, it is possible that the use of deductive qualitative analyses (mapping interview content to the CFIR) would provide additional insights and thus should be considered in future research. The comparison of high- and low-sustainability programs contributes new information to walking school bus research and can be applied to other community-based physical activity interventions and areas of implementation science. Some constructs were challenging to rate, as indicated by the low inter-rater agreement for some constructs. Reconciliation between raters was used to improve the quality of ratings, but findings for constructs with lower inter-rater agreement should be interpreted with caution. The study findings may not generalize to the experiences of all walking school bus program leaders, though participants were selected systematically from a large pool of programs from across the USA. Due to perceived difficulty in identifying and recruiting programs that had failed to be sustained, the study relied on respondents to report on their perception of whether the program was likely to be sustained, which may not always reflect reality. Future studies could follow programs over time to capture more accurate measures of sustainability and collect prospective measures of contextual factors. While the present study used an empirical approach to identifying factors related to sustainability by linking separate questions (i.e., linking questions on CFIR constructs to a question on program sustainability), other approaches may be worth exploring, such as having respondents share *their perceptions* of how specific factors (e.g., CFIR constructs) impacted or are impacting the sustainability of their program.

### Implications for practice

This and previous research on walking school bus implementation point to several recommendations for improving uptake, implementation, and sustainment of walking school bus programs. Since walking school bus programs rely on participation from students as well as coordination and day-to-day implementation from adult volunteers, students, parents, and community members should be engaged through multiple modalities and planning activities to have high levels of ongoing involvement. While walking school bus programs can be successfully implemented exclusively by parents, fostering a supportive school climate (i.e., buy in) and involvement from school personnel are likely to increase long-term success. Clearly communicating program benefits to school personnel through reflection and evaluation, particularly those benefits that align with the mission of the school and community, appears to be a promising starting point for improving school and community buy in. Additionally, numerous ongoing activities are required to sustain walking school bus programs across school years. These activities should involve coordinated engagement efforts with multiple levels of stakeholders including families, volunteers, and school members. Since transitioning to new coordinators and/or support members over time can be challenging, establishing protocols and facilitation materials should be explored to soothe such transitions.

### Conclusions and future directions

This study was among the first to systematically examine implementation contextual factors related to community-based active travel to school interventions, particularly with regard to barriers to sustainability and strategies for overcoming these barriers. Qualitative data provided by existing program leaders pointed to promising implementation strategies for improving the success and sustainability of walking school bus programs. Future experimental research should focus on testing specific, scalable approaches for incorporating these lessons learned into implementation interventions to ultimately improve uptake and continued use of effective active travel to school interventions across the USA and world. Increasing rates of active travel to school through walking school bus programs and related efforts has potential for supporting health through promoting physical activity among large numbers of youth and even adults.

## Data Availability

The datasets used and/or analyzed during the current study are available from the corresponding author on reasonable request.
